# A Deep Convolutional Neural Network for Pneumonia Detection in X-ray Images with Attention Ensemble

**DOI:** 10.3390/diagnostics14040390

**Published:** 2024-02-11

**Authors:** Qiuyu An, Wei Chen, Wei Shao

**Affiliations:** 1 School of Computer Science, Nanjing University of Posts and Telecommunications, Nanjing 210023, China; chenwei@njupt.edu.cn; 2 Nanjing University of Aeronautics and Astronautics Shenzhen Research Institute, Shenzhen 518067, China

**Keywords:** deep learning, attention mechanism, AI in healthcare, pneumonia detection, X-ray image analysis, medical data mining

## Abstract

In the domain of AI-driven healthcare, deep learning models have markedly advanced pneumonia diagnosis through X-ray image analysis, thus indicating a significant stride in the efficacy of medical decision systems. This paper presents a novel approach utilizing a deep convolutional neural network that effectively amalgamates the strengths of EfficientNetB0 and DenseNet121, and it is enhanced by a suite of attention mechanisms for refined pneumonia image classification. Leveraging pre-trained models, our network employs multi-head, self-attention modules for meticulous feature extraction from X-ray images. The model’s integration and processing efficiency are further augmented by a channel-attention-based feature fusion strategy, one that is complemented by a residual block and an attention-augmented feature enhancement and dynamic pooling strategy. Our used dataset, which comprises a comprehensive collection of chest X-ray images, represents both healthy individuals and those affected by pneumonia, and it serves as the foundation for this research. This study delves deep into the algorithms, architectural details, and operational intricacies of the proposed model. The empirical outcomes of our model are noteworthy, with an exceptional performance marked by an accuracy of 95.19%, a precision of 98.38%, a recall of 93.84%, an F1 score of 96.06%, a specificity of 97.43%, and an AUC of 0.9564 on the test dataset. These results not only affirm the model’s high diagnostic accuracy, but also highlight its promising potential for real-world clinical deployment.

## 1. Introduction

Pneumonia continues to be a formidable public health concern as it significantly impacts global morbidity and mortality rates. The World Health Organization recognizes pneumonia as a leading cause of mortality, predominantly among children and the elderly [1]. While prompt and accurate diagnosis is critical for effective intervention, conventional diagnostic methods are hampered by their inherent limitations: they are resource-intensive, time-consuming, and heavily reliant on the availability of specialized radiological expertise.

Recent years have witnessed a paradigm shift in medical image analysis with the advent of deep learning [2,3,4,5]. Deep convolutional neural networks (CNNs)—known for their proficiency in image classification, segmentation, and feature extraction—have been at the forefront of advancing automated medical imaging systems. The application of deep CNNs for pneumonia detection has shown promise [6,7,8,9], and a potential for the rapid and autonomous identification of pulmonary infections has been suggested, which could revolutionize patient care.

However, despite these advancements, current models often fall short in adequately addressing the multifaceted challenges of pneumonia image classification. Issues such as data imbalance, inconsistent imaging standards, and the nuanced nature of pneumonia features, which often mimic other lung conditions, persist [10]. Many existing models lack the sensitivity to differentiate subtle yet critical pathological patterns, thereby leading to potential misdiagnoses [11]. Additionally, the black box nature of many deep learning models raises concerns about interpretability and trust in clinical settings [12]. Moreover, the majority of models need a great deal of computing power and time to train in order to produce results that are excellent [13].

Furthermore, the majority of CNN models extract and analyze features using a single model, which may not adequately capture the intricate details of the data [14]. In addition, a great deal of models lack the flexible scaling and generalization features that are crucial for practical use [15]. On top of that, a great deal of strategies make use of attention mechanisms without taking into account the potential effects of combining several attention mechanisms [16].

In light of these gaps, this paper introduces an innovative deep convolutional neural network that utilizes an attention ensemble, thus effectively blending the strengths of EfficientNet [17] and DenseNet [18]. Our model addresses the shortcomings of the current approaches by enhancing feature extraction and focusing more precisely on pertinent regions within the images. Furthermore, our model demonstrates flexible scalability and efficiency due to its modular components and optimized structure; moreover, a satisfactory outcome can be attained in just five learning epochs. We critically assessed our model’s performance using a comprehensive dataset, and we benchmarked its results against established methodologies. Moreover, we explored the potential of our model in clinical diagnostic scenarios, underscoring our research’s aim to not only fill the existing gaps, but to also provide a reliable, interpretable, and clinically relevant tool for pneumonia detection.

The contributions of this study can be encapsulated by the enhanced attention mechanisms introduced. Our model incorporates a sophisticated attention ensemble, including multi-head self-attention, cross-attention-based feature fusion, attention augmentation, and dynamic attention pooling. These enhancements allow the model to focus precisely on critical regions within the images, thereby improving the accuracy of pneumonia detection. The strategic use of multiple attention mechanisms is a testament to our model’s ability to handle the complexity of medical imagery, and it helps to ensure a high degree of interpretability and reliability.

The rest of this paper is organized as follows. Section 2 describes the review of the related works. Section 3 describes the proposed methodology, including model architecture and details. In Section 4, the experiments are conducted, and the performance results are presented. In Section 5, we discuss the choice of base model, the limitations of the paper, and future works. Finally, the conclusion is presented in Section 6.

## 2. Related Work

Over the last ten years, many researchers have employed deep learning to automatically identify lung infections and other conditions from chest X-rays. A notable contribution by Rajpurkar et al. [19] set a precedent in the field by demonstrating the potential of deep learning models in detecting pneumonia from chest X-rays, and their approach outperformed traditional medical imaging techniques. Since then, the field has seen considerable advancements. The study of [20] delved into the challenges of diagnosing pneumonia, a disease that is often obscured by its similarity to other lung infections (especially in pediatric cases). Leveraging the power of an ensemble of seven pre-trained CNN models, this research innovatively enhances the diagnostic accuracy for pneumonia in children; furthermore, it achieves remarkable sensitivity and AUC metrics, as well as demonstrates the efficacy of CNN ensembles in differentiating between normal, viral, and bacterial pneumonia in chest X-ray images. In [21], the authors addressed the critical need for rapid and accurate pneumonia diagnosis, particularly for vulnerable pediatric populations. They proposed a streamlined deep learning solution utilizing MobileNet, and they achieved significant accuracy, recall, and F-score metrics. The model’s efficiency, as demonstrated through its quick training time and reduced computational demand, marks a substantial advancement in facilitating early pneumonia detection and intervention. The study of [22] addressed the challenge regarding the limited availability of annotated computed tomography (CT) scans for pneumonia diagnosis by introducing a novel three-level optimization method. This method enhances the performance of deep learning models by leveraging source domain CT data to compensate for the scarcity of labeled scans in the target domain, thus effectively downweighting low-quality source data to minimize validation loss, as well as significantly improving pneumonia detection accuracy. In [23], a novel deep learning algorithm, Pneumonia-Plus, was developed to accurately classify the different types of pneumonia using CT images. The model demonstrates an impressive diagnostic performance, with AUC values for viral, fungal, and bacterial pneumonia at 0.816, 0.715, and 0.934 respectively. In addition, it outperformed two of the three radiologists in classifying bacterial and viral pneumonia, thereby highlighting its potential to assist radiologists in reducing misdiagnosis and guiding clinical decision making effectively. The study of [24] presented a scalable and interpretable deep convolutional neural network (DCNN) aimed at automating pneumonia diagnosis from chest X-ray images. It underscored the necessity for intelligent systems to overcome the limitations of human-assisted approaches, such as expert availability and treatment costs. The proposed DCNN model excels in feature extraction and the classification of images into normal and pneumonia classes, thus demonstrating its superior performance in comparison to other state-of-the-art methodologies through rigorous evaluations on various performance metrics. In the context of distinguishing complex lung diseases, the study of [25] made a notable contribution. The aforementioned research presented a comprehensive framework for predicting lung diseases like pneumonia and COVID-19 from chest X-ray images; the framework encompassed dataset acquisition, image enhancement, precise region of interest (ROI) extraction, and robust feature extraction, which were followed by a classification approach that used advanced methods, including Artificial Neural Network (ANN), Support Vector Machine (SVM), K-Nearest Neighbors (KNN), ensemble classifiers, and a specially designed deep learning architecture based on Recurrent Neural Network (RNN) with Long Short-Term Memory (LSTM). This work demonstrates the intricate interplay of various image processing and machine learning techniques to address the critical challenge of differentiating between closely related lung conditions, thereby showcasing impressive performance in comparison to the state-of-the-art models.

The integration of attention mechanisms into deep learning models has been particularly transformative in healthcare as it has enabled precise models to focus, i.e., a focus akin to the selective concentration of human cognition, on the salient features within medical images. A general survey [26] on attention mechanisms provided an extensive overview indicating their versatility across a range of applications. A significant stride in pneumonia detection was marked by [27], which harnessed the power of transfer learning and attention mechanisms. This study integrates features from ResNet152, DenseNet121, and ResNet18 with an attention-based feature selector, which significantly enhanced the performance of computer-aided diagnostic models with impressive accuracy and precision metrics. In [28], the authors enhanced DenseNet architecture by incorporating a feature channel attention block, i.e., Squeeze and Excitation (SE), to better highlight the pneumonia features in the image maps. This method, complemented by strategic pooling and activation function choices, significantly refines feature representation by focusing the attention on lesion regions, which results in notable improvements in the classification metrics.

Deep learning feature fusion techniques have also advanced quickly in recent years, thereby highlighting their major significance. The study of [29] introduced a multi-path network: the Attention Feature Fusion VGG19 (AFF-VGG19). This network integrates attention modules, feature-fusion blocks, and baseline convolutional neural networks. This innovative network architecture is adept at extracting and mining critical features from medical images, and it demonstrates remarkable accuracy in brain tumor classification, brain disorder distinction, and dementia grading tasks on large-scale MRI datasets. In [30], the authors proposed a novel evidence-based fusion approach. Leveraging the Dempster–Shafer theory, they successfully integrated five pre-trained convolutional neural networks—including VGG16, Xception, InceptionV3, ResNet50, and DenseNet201—and they achieved remarkable pneumonia detection accuracy with a precision of 97.5%, a recall of 98%, an F1-score of 97.8%, and an accuracy of 97.3%. In addition to medical image classification, fusion techniques can also be applied to more computer vision problems. A notable instance is presented in [31], where the authors address the paucity of research in self-supervised learning (SSL) for action recognition (AR). They proposed DuTriNet, a unique model that employs a dual-stream approach to capture temporal correlations by fusing optical flow information with Weber maps within a triplet Siamese architecture. This contribution represented a significant advance in the field of AR.

All things considered, there are several deep learning-based models for pneumonia detection; the attention mechanism improves the model’s detection performance, and feature fusion methods help to further hone the methodology. However, few of the models currently in use integrate these tactics. In order to reinforce these features, we therefore suggest a deep convolutional neural network that extracts features using two base CNN models and then applies a number of attention components. Our model differs significantly from current approaches in that it combines numerous attention modules, exhibits effective learning capabilities, and requires less resources.

## 3. Methodology

**Figure 1:**
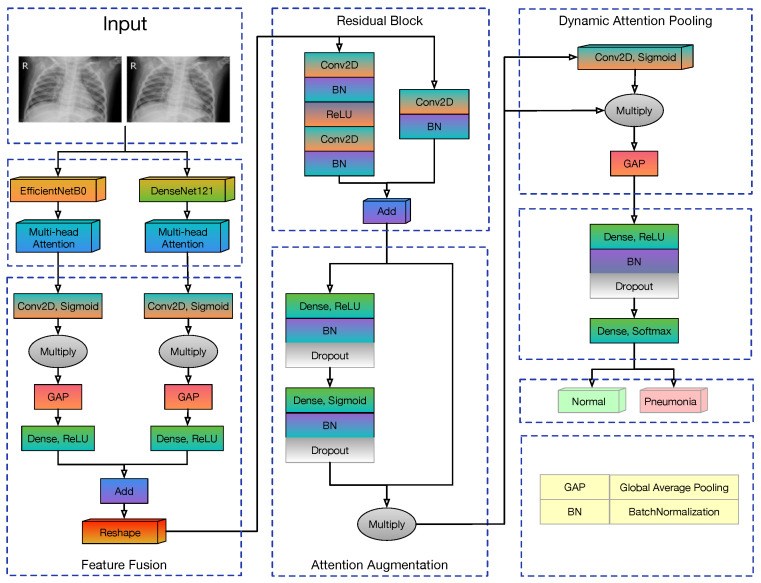
Model architecture.

In contemporary deep learning research, the fusion of information and features from different sources has been demonstrated to bolster model performance, especially in the realm of image classification. With this in consideration, we devised a deep convolutional neural network with an attention ensemble, which was aimed at effectively detecting and classifying pneumonia from chest X-ray images. The overall architecture of our model can be seen in Figure 1.

The input to our model is a chest X-ray image of size 224×224×3. These images underwent a series of preprocessing steps including scaling, normalization, and augmentation to fit the network and enhance data diversity. The output from the model is a probability distribution over two classes: normal and pneumonia. This form of output provides more insight for diagnosis beyond just a simple categorical label.

- Feature Extraction: At this point, the input image’s features are captured and are crucial for medical picture analysis. We utilized EfficientNet-B0 and DenseNet-121, two advanced architectures, to accomplish thorough feature extraction. Additionally, each feature extraction network’s output was subjected to multi-head attention techniques to improve the representation of crucial areas.
- Feature Fusion and Enhancement: Following this, the model fuses the features, thereby forming a unified feature representation. Additionally, the residual block, attention-based augmentation and dynamic pooling were incorporated to further reinforce the representational strength of the fused features.
- Classification Decision: Upon the culmination of the entire feature extraction, as well as the fusion and enhancement workflow, the model processes the information through a neural network, ultimately outputting a probability distribution over the classes of normal and pneumonia.

The structure of this model was devised to ensure that all the valuable information extracted from the image is harnessed efficiently as the aim was to strive for optimal performance in the pneumonia detection task.

### 3.1. Data Augmentation

Data augmentation is an indispensable technique in deep learning. When dealing with a limited or imbalanced dataset, the artificial expansion of the training dataset through data augmentation can greatly improve the generalization capability of the model. This is especially the case for medical images, where a significant amount of specialized data augmentation is required to avoid unrealistic situations.

In our research, the data augmentation techniques used included rotation, shift, shear, zoom, and brightness. Each technique’s parameter is shown in Table 1.

Also, we applied histogram equalization to the images to enhance their contrast.

The richness and diversity of the training dataset are augmented through a succession of data augmentation procedures. Furthermore, the data enhancement was applied at a suitable strength so as not to cause image distortion.

### 3.2. Feature Extraction

#### 3.2.1. Base Network

Medical images, such as chest X-rays, inherently possess rich information within them. Intricate details like shapes, textures, and patterns are essential for medical image classification. To effectively capture these intricate details in X-ray images, we leveraged two powerful architectures: EfficientNet and DenseNet.

1. EfficientNetB0: EfficientNet is a family of models designed for scaling neural network architectures, and it is renowned for achieving state-of-the-art accuracy with fewer parameters. The architecture utilizes compound scaling, where the depth, width, and resolution of the network are scaled in a coordinated manner. For our task, we employed the EfficientNetB0 variant.
2. DenseNet121: Dense convolutional network (DenseNet) leverages inter-layer connectivity patterns that promote feature reuse and reduce the risk of overfitting. Each layer in DenseNet obtains additional inputs from all preceding layers and passes its feature maps to all subsequent layers. This intricate connectivity ensures that the network captures a wide range of spatial features, thus making it particularly suitable for our dataset. In our research, we chose DenseNet121 as one of the base model.

With the combined power of EfficientNetB0 and DenseNet121, our model was equipped to capture a comprehensive understanding of the input image, thus paving the way for accurate and reliable disease classification.

#### 3.2.2. Multi-Head Attention Mechanism

The attention mechanism has revolutionized, primarily in natural language processing, the way through which deep learning models interpret sequential data. Its power resides in its capability to prioritize specific parts of the input data, whereby it focuses more on information that is more relevant for the task at hand. In the context of our image recognition task, this capability was found to be pivotal in the highlighting of regions in the X-ray images that are more indicative of pneumonia.

The essence of the attention mechanism lies in its weighted context calculations. For any given input, the attention model computes a set of attention scores. These scores determine how much importance should be given to each part of the input when producing an output. Mathematically, this can be expressed as follows:

(1) $$\mathrm{Attention}\left(Q,K,V\right)=\mathrm{softmax}\left(\frac{QK^{T}}{\sqrt{d_{k}}}\right)V,$$

where *Q*, *K*, and *V* are the query, key, and value matrices, respectively, and $d_{k}$ is the dimension of the key.

Multi-head attention, an extension of the basic attention mechanism, splits the input into multiple parts (heads) and applies attention to each head separately. The outputs from each head are then concatenated and linearly transformed, thereby providing a diverse set of attention patterns.

We leveraged the transformer MultiHeadAttention layer from Keras to enhance the outputs of both EfficientNetB0 and DenseNet121 with attention. By doing so, we ensured that the model gives prominence to regions in the X-ray image that contain critical indicators of pneumonia. The main parameters employed for this attention mechanism are elaborated below:

- **Number of Heads (num_heads):** We opted for 4 heads as this signified that the attention mechanism can concurrently take into account 4 different weight distributions. This aids the model in capturing dependencies in features at various levels.
- **Dimension of Keys (key_dim):** The dimension of the keys was set at 128. This parameter dictates how the attention mechanism weighs the input information. A larger key dimension facilitates the model to consider a richer set of information when computing attention weights.

By integrating this attention mechanism during the feature extraction phase, the model can more precisely focus on those image regions that are crucial for the classification decision, thus potentially enhancing the accuracy for pneumonia detection.

### 3.3. Feature Fusion and Enhancement

The comprehensive procedure of feature fusion and enhancement in our proposed model is one of the pivotal contributions of this work. It consists of distinct stages that were meticulously designed to amplify the discriminative capacity of the learned feature representations. This synergy is crucial for enhancing the model’s ability to detect and analyze pneumonia indicators from chest X-rays effectively.

#### 3.3.1. Feature Fusion

Feature fusion serves as the cornerstone of our model, where the feature maps are synergistically combined to harness the strengths of each base network. It uses attention mechanisms to refine the integration process, thereby ensuring that the fused features are optimally aligned for the task at hand.

- Channel-attention mechanisms were used to compute the attention weights for each feature map.
- Attention-weighted feature maps from EfficientNetB0 and DenseNet121 were element-wise multiplied for selective feature enhancement.
- The features were then projected onto a common feature space to normalize the channel dimensions. The quantity of neurons in the dense layer was defined by the subsequent reshaping process. To ensure that the reshaped features preserved the input’s physical characteristics to the greatest extent, we opted for a 7 × 7 dimension configuration.
- The resulting feature maps were added to form a fused feature map.
- The enhanced features were reshaped to fit the subsequent layers of the network architecture.

Algorithm 1 outlines this process.
**Algorithm 1** Feature Fusion Function  1:**function** FeatureFusion($F_{eff},F_{dense}$)  2:      $F_{eff\_att}\leftarrow$Conv$\left(F_{eff_{c}hannels},\left(1,1\right){,}^{\prime }sigmoid^{\prime }\right)\left(F_{eff}\right)$  3:      $F_{dense\_att}\leftarrow$Conv$\left(F_{dense_{c}hannels},\left(1,1\right){,}^{\prime }sigmoid^{\prime }\right)\left(F_{dense}\right)$  4:      $F_{eff}\leftarrow F_{eff}\cdot F_{eff\_att}$  5:      $F_{dense}\leftarrow F_{dense}\cdot F_{dense\_att}$  6:      $F_{eff}\leftarrow$GlobalAveragePooling$\left(F_{eff}\right)$  7:      $F_{dense}\leftarrow$GlobalAveragePooling$\left(F_{dense}\right)$  8:      $F_{eff\_mapped}\leftarrow$Dense$\left(784{,}^{\prime }relu^{\prime }\right)\left(F_{eff}\right)$  9:      $F_{dense\_mapped}\leftarrow$Dense$\left(784{,}^{\prime }relu^{\prime }\right)\left(F_{dense}\right)$10:      $F_{eff\_mapped}\leftarrow$Dropout$\left(0.23\right)\left(F_{eff\_mapped}\right)$11:      $F_{dense\_mapped}\leftarrow$Dropout$\left(0.23\right)\left(F_{dense\_mapped}\right)$12:      $F_{fused}\leftarrow F_{eff\_mapped}+F_{dense\_mapped}$13:      $F_{reshaped}\leftarrow$Reshape$\left(\left(7,7,16\right)\right)\left(F_{fused}\right)$14:      **return** $F_{reshaped}$15:**end function**

In this model, achieving a balanced integration of features from both networks is essential. Our approach ensures that the unique strengths of EfficientNet and DenseNet are not just preserved, but also synergistically combined. This results in a rich and comprehensive spatial feature set that significantly enhances the model’s capability in detecting and analyzing pneumonia from X-ray images.

#### 3.3.2. Residual Block

A custom residual block was then applied to the fused features, whereby the aim was to strengthen feature propagation and alleviate the vanishing gradient problem. The residual block follows the standard architecture of convolutional layers with batch normalization and ReLU activations, including a shortcut connection for identity mapping. In this, we set 256 filters in the convolutional layers.

#### 3.3.3. Attention Augmentation

Subsequent to residual integration, an attention augmentation mechanism was introduced. Attention augmentation further refined the feature representation by imposing an additional attention mechanism, which selectively emphasizes salient features and suppresses irrelevant ones. It involves the following steps:

- A dense layer, which is followed by batch normalization and dropout, generates an attention map from the concatenated features.
- The attention map undergoes a sigmoid activation, batch normalization, and another dropout to stabilize the training.
- The attention map is then applied to the concatenated features to yield attention-weighted features.

The pseudocode for attention augmentation is demonstrated in Algorithm 2.
**Algorithm 2** Attention Augmentation Function  1:**function** AttentionAugmentation(*F*)  2:      A←Dense$\left(F_{channels}{,}^{\prime }relu^{\prime }\right)\left(F\right)$  3:      A←BatchNormalization(A)  4:      A←Dropout(0.23)(A)  5:      A←Dense$\left(F_{channels}{,}^{\prime }sigmoid^{\prime }\right)\left(A\right)$  6:      A←BatchNormalization(A)  7:      A←Dropout(0.23)(A)  8:      $F_{att\_weighted}\leftarrow F\cdot A$  9:      **return** $F_{att\_weighted}$10:**end function**

The attention fusion can be summarized by the following:

(2) $$F_{attention}=A⊙F_{fused},$$

where *A* represents the attention map and ⊙ denotes the Hadamard product, thereby ensuring each feature in $F_{fused}$ is scaled according to its significance.

#### 3.3.4. Dynamic Attention Pooling

Traditional pooling layers in convolutional neural networks serve to reduce the spatial dimensions of feature maps by aggregating the information. However, these methods often employ static operations, like average or max pooling, which may result in a loss of critical details. The idea behind dynamic attention pooling is to allow the model to adaptively decide the regions of importance in the feature map before pooling, thus preserving crucial information.

Dynamic attention pooling concludes the feature enhancement pipeline by applying a spatial attention mechanism that allows the model to focus adaptively on the most informative parts of the feature map. It involves the following steps:

- Generate a spatial attention map via a convolution layer with a sigmoid activation.
- Multiply the original feature map by the attention map to obtain a weighted feature map.
- Perform global average pooling on the weighted feature map to extract the pooled feature descriptor.

Formally, the dynamic attention pooling can be depicted as follows:

(3) $$P_{dynamic}=A_{pool}⊙Pool\left(F_{attention}\right),$$

where $A_{pool}$ is the attention map for pooling and Pool is the traditional pooling operation (e.g., global average pooling). Here, $A_{pool}$ dynamically adjusts the influence of each spatial location during pooling.

The process of dynamic attention pooling is shown in Algorithm 3.
**Algorithm 3** Dynamic Attention Pooling Function1:**function** DynamicAttentionPooling(*F*)2:      $A_{map}\leftarrow$Conv2D$\left(1,\left(1,1\right){,}^{\prime }sigmoid^{\prime }\right)\left(F\right)$3:      $F_{weighted}\leftarrow F\cdot A_{map}$4:      $F_{pooled}\leftarrow \mathrm{GAP}\left(F_{weighted}\right)$5:      **return** $F_{pooled}$6:**end function**

### 3.4. Classifier

In the final layer of our model, a strategic sequence of operations was implemented to refine the decision-making process. Initially, the input was passed through a fully connected dense layer with 128 neurons by employing the ‘relu’ activation function to introduce non-linearity, which is crucial for learning complex patterns. Furthermore, L2 regularization was applied to mitigate overfitting via penalizing large weights.

Subsequently, a batch normalization layer was applied. This layer normalizes the activations from the previous layer via addressing internal covariate shifts by stabilizing the learning process and expediting convergence.

Following batch normalization, a dropout layer was introduced. Dropout is a powerful regularization technique that prevents overfitting by randomly setting a fraction of input units to zero at each update during training time, which helps to make the model more robust.

The classification culminated with the implementation of another dense layer that comprised 2 neurons corresponding to the number of classes, which was achieved utilizing the ‘softmax’ activation function. This function converts the output to a probability distribution, which allowed the model to make a multi-class prediction, thus effectively categorizing the input into one of the two possible classes.

The detailed process of classification is shown in Algorithm 4.
**Algorithm 4** Neural Network Classifier Layers1:x←Dense(128,‘relu’,kernel_regularizer=l2(0.006))2:x←BatchNormalization3:x←Dropout(0.23)4:output←Dense(2,‘softmax’)

## 4. Experiments

In the experiments, we trained our proposed model with the Adam optimizer and implemented a warmup learning rate schedule to ensure smooth optimization. We chose categorical cross-entropy as the loss function, reflecting our focus on multi-class classification. The performance of the model was rigorously evaluated across several metrics, including accuracy, F1 score, AUC, and specificity, to ensure a comprehensive assessment of its predictive power and reliability.

The experiments were run on a workstation equipped with an NVIDIA RTX 4090 GPU, 24 GB VRAM, and a 14-core AMD EPYC 7453 CPU with 64 GB system memory; this provided substantial computational resources for model training and inference. The software environment consisted of Python 3.10.12 and TensorFlow 2.13.0, which enabled efficient utilization of the GPU for deep learning through CUDA and cuDNN integration.

This section commences with a detailed description of the dataset employed in the experiment. Additionally, it delineates the evaluation metrics that were utilized for assessing the model’s performance, which was followed by an analytical discussion of the hyperparameters optimized for the experimental setup. Subsequently, we elucidate on the experimental outcomes and provide a comprehensive analysis of these results. To contextualize our model’s performance, we conclude by drawing comparisons with established baseline models, as well as with contemporary state-of-the-art models.

### 4.1. Dataset Description

The dataset utilized for this study was sourced from the Guangzhou Women and Children’s Medical Center, Guangzhou, and it has been made publicly available on Kaggle. Prior to the commencement of the analysis, images of substandard quality or those that were illegible were excluded. The integrity of the remaining images was then assessed and classified by two experienced physicians [32].

Comprising a total of 5856 JPEG images, the dataset was categorized into two distinct classes: pneumonia and normal. It was also systematically organized into three separate directories: training, testing, and validation. Each directory was further partitioned into subdirectories representing the two diagnostic categories. Specifically, the training directory housed 5216 images, with 3875 labeled as pneumonia and 1341 as normal. The testing directory contained 624 images, which were split into 390 pneumonia and 234 normal. The validation directory was composed of a modest collection of 16 images, and these were evenly distributed with 8 pneumonia and 8 normal images.

Despite the variance in image sizes, all of the images were of superior quality and were uniformly resized to a standard dimension prior to their incorporation into the training process. The pneumonia category outnumbered the normal category, which introduced a potential bias. To rectify this imbalance, category-specific weights were computed and applied during the training phase to ensure a balanced representation and fair assessment by the model.

**Figure 2:**
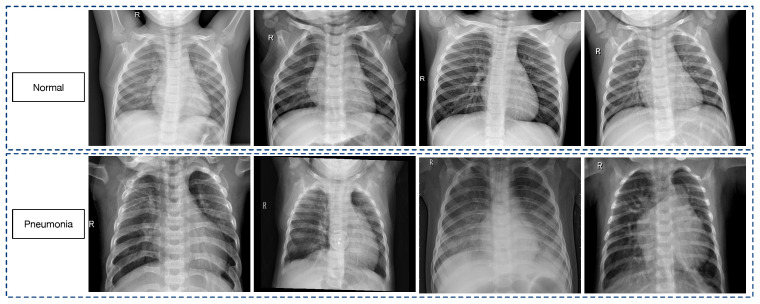
Some samples of the chest X-ray images dataset.

Some samples of the images are shown in Figure 2.

### 4.2. Evaluation Metrics

To evaluate the performance of our model, we utilized a confusion matrix, which is a table used to describe the performance of a classification model on a set of test data for which the true values are known. The matrix compares the actual target values with those predicted by the model, thus providing insight into the correctness of the classifications made. The terms used in the confusion matrix were as follows: true positives (*TP*), true negatives (*TN*), false positives (*FP*), and false negatives (*FN*).

Based on the confusion matrix, the following metrics were calculated:

- Accuracy: The ratio of correctly predicted observations to the total observations. It is given by Equation (4):
  (4) $$\mathrm{Accuracy}=\frac{TP+TN}{TP+TN+FP+FN}.$$
- Precision: The ratio of correctly predicted positive observations to the total predicted positive observations. Precision is defined in Equation (5):
  (5) $$\mathrm{Precision}=\frac{TP}{TP+FP}.$$
- Recall: Also known as sensitivity, it measures the ratio of correctly predicted positive observations to all of the observations in the actual class. The recall is calculated as shown in Equation (6):
  (6) $$\mathrm{Recall}=\frac{TP}{TP+FN}.$$
- F1 Score: The weighted average of precision and recall. Therefore, this score takes both false positives and false negatives into account. It is computed with Equation (7):
  (7) $$F1\;\mathrm{Score}=2\times \frac{\mathrm{Precision}\times \mathrm{Recall}}{\mathrm{Precision}+\mathrm{Recall}}.$$
- Specificity: The ratio of correctly predicted negative observations to the all of the observations in the actual class. Specificity is detailed in Equation (8):
  (8) $$\mathrm{Specificity}=\frac{TN}{TN+FP}.$$
- AUC: The area under the receiver operating characteristic (ROC) curve is a performance measurement for classification problems at various threshold settings. The AUC represents the degree of separability between classes, and is represented as a value between 0 and 1.

These metrics collectively provide a comprehensive understanding of the model’s performance beyond mere accuracy, and this is achieved by considering the balance between sensitivity and specificity, as well as the trade-offs between precision and recall.

### 4.3. Training Hyperparameters

The efficacy and performance of machine learning models are profoundly influenced by the selection of hyperparameters. Hyperparameters, unlike model parameters, are set prior to the commencement of the training process and govern the overall behavior of the model. They play a crucial role in controlling the dynamics of the learning algorithm, impacting factors such as the speed of convergence to the global minimum, the model’s ability to generalize from the training data to unseen data, and the prevention of overfitting.

In recognizing the pivotal role of hyperparameters, we employed Optuna, a state-of-the-art hyperparameter optimization framework, to systematically explore and identify the optimal hyperparameter values for our experiment [33]. Optuna’s efficient search capability streamlined the trial-and-error process and converged on the best results within the multidimensional hyperparameter space.

In the pursuit of optimal hyperparameters for our model, we leveraged Optuna with its tree-structured Parzen estimator (TPE) algorithm to conduct our search. We configured Optuna to perform 20 trials. Each trial represented a complete training process of the model using a unique set of hyperparameters that were suggested by the TPE algorithm. By evaluating the model’s performance after each trial, Optuna and the TPE algorithm refined their understanding of the hyperparameter space by focusing on the subsequent searches around the most promising regions. The following hyperparameters were optimized, as shown in Table 2.

**Figure 3:**
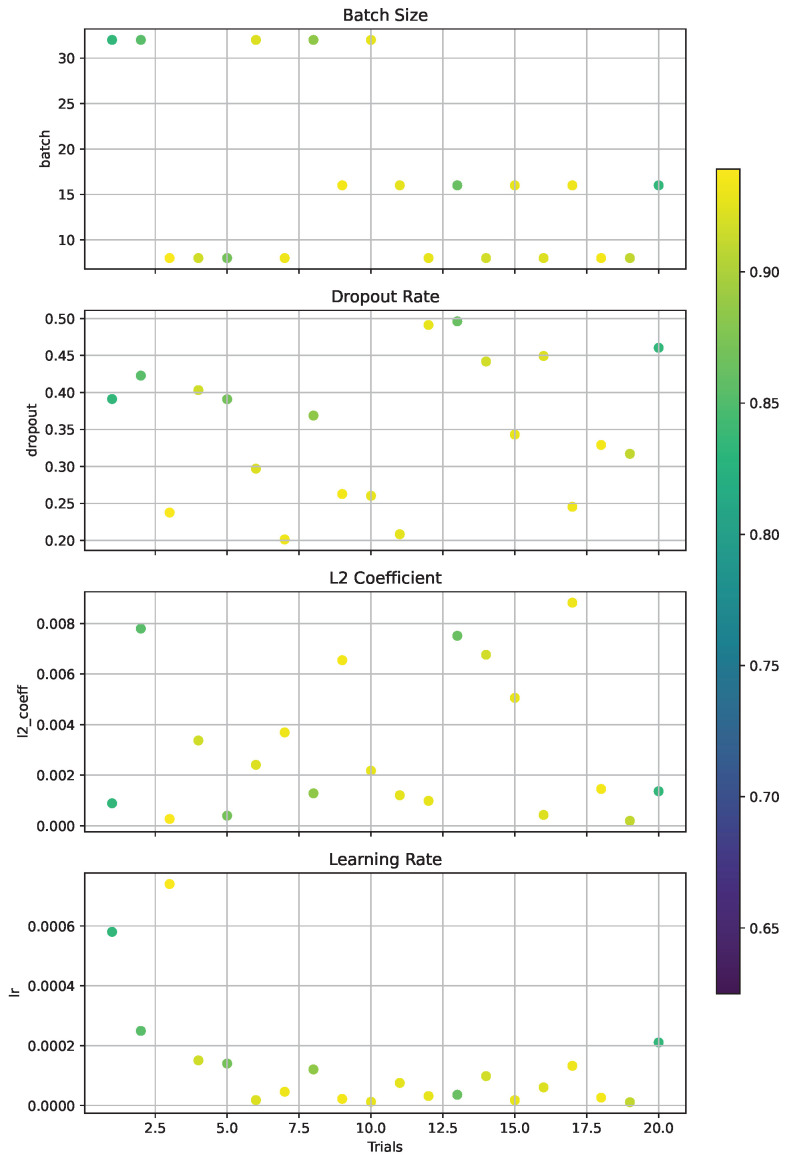
Hyperparameter sensitivity analysis for model accuracy post-optimization with Optuna.

The provided image (Figure 3) illustrates the intricate relationships between the four hyperparameters—batch size, dropout, L2 coefficient, and learning rate—and the model’s accuracy. This visual representation, which was generated post-optimization by Optuna, offered valuable insights into the model’s behavior and sensitivity to hyperparameter values.

- For the batch size, we observed that smaller batch sizes generally correlate with higher accuracy. This trend suggests that smaller batches provide a regularizing effect and a more robust gradient estimation, which might help the model avoid local minima during training.
- The dropout hyperparameter plot indicates a less clear-cut relationship. While dropout rates around 0.25 seem to yield higher accuracy, thereby suggesting that this level of regularization is beneficial in preventing overfitting, the spread of the lines implied that the model’s accuracy is relatively stable across a range of dropout values.
- The L2 coefficient graph shows a tendency toward smaller coefficients correlating with higher accuracy. This relationship highlights the importance of regularization in reducing overfitting, but it also suggests that too much penalization on the weights can hinder the model’s ability to learn complex patterns.
- The learning rate graph displays a distinct pattern where very low learning rates are associated with higher accuracy. This emphasizes the need for a sufficiently small learning rate to allow the model to converge gradually and to avoid overshooting the minimum of the loss function.

These visualizations underscore the delicate balance that must be struck when choosing hyperparameters. They reflect the model’s complex dynamics and emphasize the necessity of a methodical approach, such as the one provided by Optuna, to navigate the hyperparameter space effectively. The chosen hyperparameters after optimization appear to be well tuned to the model’s architecture and data, as evidenced by the observed accuracy levels.

### 4.4. Results and Analysis

The model’s performance was first evaluated on an independent test set following the general training process. The training process spanned 5 epochs, with each epoch taking approximately 82–96 s. The total training time for the entire model training process was 425 s. The results of this initial assessment are documented in Table 3.

Moreover, to ensure the robustness and generalizability of our model, we performed a stratified five-fold cross-validation on the training dataset. In this process, the dataset was divided into five equal parts, with each fold serving as the validation set once while the remaining four folds were used for training. This technique allowed for a comprehensive evaluation of the model’s performance across the entire dataset. The cross-validation results are also presented in Table 3, and they showcase the mean and standard deviation of the metrics obtained from the folds.

The test results demonstrated an impressive accuracy of 95.19%, with the precision reaching a remarkable 98.38%. This precision was particularly noteworthy as it suggests the model’s effectiveness in identifying true positive cases, which is a critical aspect in many practical applications where the cost of false positives is high. An F1 score of 96.06% reflects a harmonious balance between precision and recall. An AUC of 0.9564 and a specificity of 97.43% further reflect the model’s capability in discerning the classes accurately and its robustness against false positives.

During the five-fold cross-validation process, our model demonstrated robustness with an average accuracy of 91.30%. Notably, the model exhibited an exceptional precision of 98.38%, with minimal fluctuation across the different folds, which indicates its consistent performance in identifying true positive cases of pneumonia. The recall rate displayed more variation, averaging at 89.34% with a standard deviation of 9.81%. This level of recall, while slightly variable, still indicated a commendable sensitivity of the model to true positive cases. The F1 score, which harmonizes precision and recall, stood at a solid 93.56% with a standard deviation of 5.19%. This balance is crucial in a medical context where both identifying the condition and minimizing false diagnoses are equally important.

Particularly commendable were the specificity and AUC metrics, which maintained high values throughout the validation process. The model exhibited a specificity of 96.94% ± 4.58% in a metric that is pivotal in medical diagnostics for its role in reducing false positives—a high specificity rate implies the model’s adeptness at correctly identifying cases without conditions. This level of specificity is essential in a clinical context as it could potentially reduce the number of unnecessary treatments, thus sparing patients from undue stress and healthcare systems from incurring extra costs.

Furthermore, the AUC score, which stood robustly at 0.9314 ± 0.0385, underscores the model’s capacity to distinguish with precision between the presence and absence of disease. An AUC score close to 1.0 is indicative of excellent model performance, and—in this context—it demonstrates the model’s strong discriminative ability. In practical terms, this high AUC score means the model is highly capable of differentiating between healthy individuals and those with pneumonia, which is of utmost importance in a field where accuracy can have a direct impact on patient outcomes.

Achieving such reliable and consistent results—particularly in terms of precision and specificity—within the constrained training duration of just five epochs, emphasizes not only the model’s efficacy, but also its practical applicability. It is well suited for deployment in real-world clinical settings, where timely and accurate diagnosis is crucial.

The performance of the proposed model and its variants, along with the baseline models, is summarized in Table 4. The proposed model demonstrated outstanding performance metrics, thus signifying the effectiveness of its architecture and the contribution of each component.

The ablation studies revealed the impact of each component on the model’s performance. The removal of the feature fusion component, which was replaced with a simple fusion strategy (i.e., dimensionality reduction followed by concatenation), caused a noticeable decline in the accuracy and specificity, thus emphasizing its role in feature integration. This may be due to the loss of the enhancement of channel attention for the features to be fused when the model fusion strategy is replaced with a simple fusion strategy. The attention augmentation component significantly contributed to recall, thereby suggesting its importance in capturing relevant features. Furthermore, the absence of dynamic pooling led to a substantial drop in specificity, which underscores its utility in spatial feature representation. The multi-head attention component’s removal decreased precision, thus indicating its effectiveness in focusing on pertinent aspects of the data. The combined absence of all the components resulted in diminished performance across all metrics, which highlighted the synergetic effect of the components.

Furthermore, our investigation yielded a notable observation: the model’s recall metrics experienced an enhancement upon the exclusion of feature fusion, attention augmentation, dynamic pooling, and multi-head attention. This occurred despite a subsequent downturn in the model’s comprehensive performance. We discerned that each of these components operates with a distinct attention mechanism. Yet, when these modules are collectively removed, which leads to the model’s minimal reliance on attention mechanisms, there is an observable decline in the model’s overall efficacy. This pattern held consistent across both baseline models. A thorough exploration of this phenomenon is reserved for discussion in Section 5.2.2.

### 4.5. Comparative Analysis

#### 4.5.1. Comparative Analysis of the Baseline Models

We first present a comprehensive comparison of the proposed model against a suite of renowned baseline models to benchmark its performance. The comparative analysis is summarized in Table 5, which provides a quantitative evaluation across several key metrics.

The proposed model outshone the baseline models across the board, particularly in terms of precision and specificity, which are critical for reducing false positives in practical applications. Its remarkable AUC of 0.9564 indicates a superior ability to discriminate between classes, which is a testament to the model’s robustness. Furthermore, the proposed model achieved these results within a time frame comparable to that of the baselines, which is noteworthy considering it only required five epochs to attain this level of accuracy. This efficiency was particularly significant as it implies less computational demand and reduced time with respect to deployment in real-world scenarios.

EfficientNetB0, with its compound scaling methodology, excels in recall, thus suggesting that its depth, width, and resolution scaling strategy is effective at identifying positive cases. However, it lacks precision and specificity, areas where our proposed model excels by leveraging an attention mechanism that EfficientNetB0 lacks. The proposed model benefits from a similar scaling approach but further refines feature extraction with attention modules to reduce false positives.

DenseNet121’s close performance in specificity and F1 score indicates that its densely connected layers excel at feature preservation—a concept we have extended in our model through attention-based feature fusion. This connection underscores the effectiveness of the dense connectivity in both our model and DenseNet121 for capturing the discriminative features necessary for accurate classification.

VGG16, while not as advanced, demonstrates the importance of depth in a network with its successive convolutional layers. The high recall indicates its potential to detect pneumonia cases, but the low specificity and AUC reflect its limitations in differentiating fine-grained features. Our proposed model addresses this with the use of the feature fusion strategy, which allows for a more nuanced analysis, similar to what VGG16 attempts to achieve, with depth.

ResNet50 and InceptionV3’s balanced performances can be attributed to their skip connections and mixed-level feature concatenation, respectively. These architectural choices are somewhat mirrored in our model, where residual connections and multi-level feature aggregation are employed to enhance learning. The multiple attention mechanisms in our model draw inspiration from the strengths of these architectures and extend their capabilities to focus on processing the most informative features.

MobileNetV3’s lower performance is indicative of the trade-offs inherent in a lightweight architecture. It suggests that, while such models are designed for efficiency, they may not capture the complexity of medical images effectively. Our model takes this into account by maintaining efficiency in computation without compromising on the depth and complexity needed for accurate medical image classification.

#### 4.5.2. Comparative Analysis of Recent Models

In the rapidly advancing field of medical image analysis, several studies have proposed deep learning models for the detection of pneumonia from chest X-ray images. We now present a comparative analysis of our proposed model against these recent advancements.

- Shagun Sharma and Kalpna Guleria [34] proposed a model that leverages VGG-16 and neural networks to enhance the detection and classification of pneumonia, thus producing a robust model with a strong precision measure.
- Harsh Bhatt [35] developed a convolutional neural network ensemble model that showcased the ability to achieve high recall values, which is crucial for medical diagnostic systems, where missing a positive case can be detrimental.
- Shimpy Goyal [25] presented a framework that incorporates deep learning and machine learning techniques to differentiate pneumonia from other lung diseases, including COVID-19, and they achieved substantial accuracy.
- Alhassan Mabrouk [36] introduced an ensemble model of deep convolutional neural networks, thereby achieving high F1 scores that are indicative of a balanced precision–recall trade-off.
- K. Wang and P. Jiang [28] enhanced DenseNet architecture by incorporating a feature channel attention block, i.e., Squeeze and Excitation (SE), to better highlight the pneumonia features in the image maps.

The performance metrics of these models along with our proposed model are tabulated in Table 6.

Our proposed model exhibits superior accuracy and precision compared to the referenced models, which is indicative of its effectiveness in correctly identifying cases of pneumonia. Notably, a precision of 98.38% underscored the model’s capability to minimize false positives, which is a critical aspect in medical diagnostics. A recall of 93.84%, while slightly lower, was complemented by the model’s high precision, thereby resulting in an F1 score that balanced the two metrics effectively. Our model’s performance was achieved within a short training period of only five epochs, which reflected its efficiency and the practical applicability of the training methodology.

In contrast, while Bhatt’s model attained the highest recall, it did so at the cost of a lower precision, which could result in a higher number of false positives. Goyal’s model presented a balanced recall and F1 score but did not reach the precision levels of our proposed model. Mabrouk’s approach showed comparable performance across all metrics, thus indicating a well-rounded model but with slightly lower overall scores than our proposed model. Wang’s method exhibited relatively high recall scores, but the overall metrics were not as good as our proposed model.

In conclusion, our proposed model not only demonstrates outstanding performance in accuracy and precision, but it also achieves these results efficiently and requires fewer epochs for training. This efficiency, combined with the model’s superior metrics, positions it as a highly competitive solution in the field of pneumonia detection from chest X-ray images.

## 5. Discussion

### 5.1. Selection of the Base Model

The architecture of our proposed model is predicated on the synergistic integration of two base models for initial feature extraction—EfficientNet and DenseNet. The rationale behind this choice is rooted in theoretical and empirical considerations. EfficientNet, known for its scalability and efficiency, provides a compact yet powerful framework for extracting high-level features, while DenseNet offers excellent feature propagation through its densely connected layers and ensures maximum information flow between the layers in the network.

This composite approach is designed to leverage the strengths of both architectures, and it was hypothesized that such an amalgamation would yield a rich and diverse feature set that is conducive to accurate pneumonia detection from chest X-ray images. The feature fusion step was critical as it harmonizes the extracted features into a cohesive representation, thereby priming them for the subsequent enhancement and classification phases illustrated in the model’s schematic.

**Figure 4:**
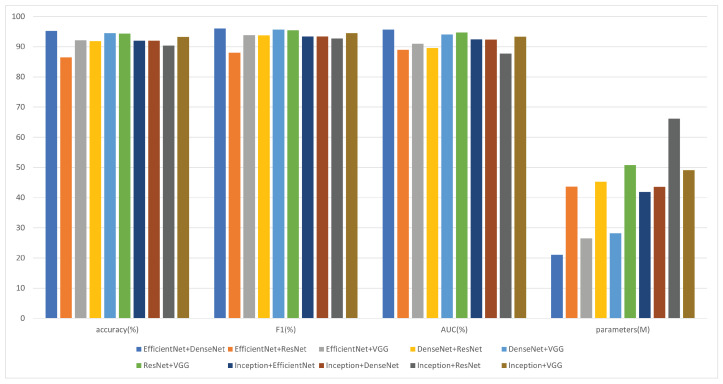
Comparative performance of the different base model combinations.

The experimental results, as depicted in Figure 4, validate the effectiveness of the chosen combination.

The model that employed EfficientNet and DenseNet outperformed the other combinations achieving the highest accuracy and F1 score, alongside a superior AUC of 0.9564. Notably, this combination also maintained a lower parameter count, which translated to a more efficient model in terms of computational resources.

While other combinations like DenseNet+VGG and ResNet+VGG showed promising results, they did not reach the efficiency and efficacy of the EfficientNet+DenseNet pairing. The EfficientNet+ResNet combination, despite its theoretical appeal, fell behind in performance, thus suggesting that the specific characteristics of EfficientNet and DenseNet are more complementary for the task at hand.

In essence, the empirical evidence strongly supported our theoretical premise in selecting EfficientNet and DenseNet as the foundational building blocks of our model. This selection not only capitalized on the inherent advantages of both architectures, but it also aligned perfectly with the model’s overall design philosophy, i.e., the aim for high accuracy with computational efficiency. Our analysis and experiments in this section show that a single model that performs well does not necessarily show good results when combined, and the combination of EfficientNet and DenseNet was found to be an optimal choice in terms of overall performance and computational efficiency.

### 5.2. Limitations

#### 5.2.1. Feature Reshaping and Spatial Information Loss

The first limitation arises from the feature fusion stage, where the global average pooling (GAP) and dense layers were employed, and this was followed by a reshaping operation that aims to align the feature maps for subsequent layers. While this maintains the dimensionality of the feature maps, it may inadvertently lead to a loss of critical spatial information that encodes the locational attributes of pathological features within the chest X-rays. This was posited based on the theoretical understanding that such operations, although preserving the depth of feature channels, can dilute the precise spatial relationships, as might be observable in a Gradient-weighted Class Activation Mapping (GradCAM) analysis.

**Figure 5:**
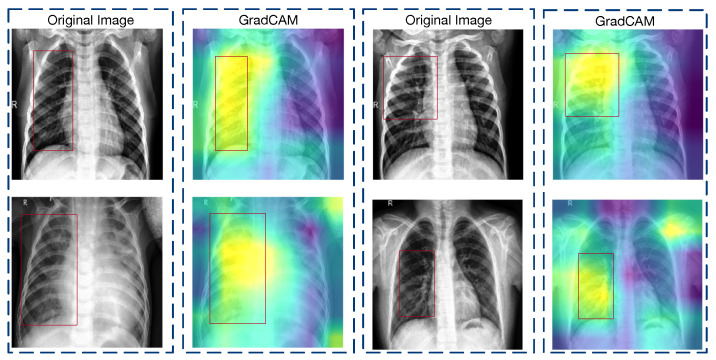
GradCAM visualization of the feature fusion pre-processing layers, where the focus is on the multiplication operations prior to the global average pooling and dense layers. Activation areas are more clearly concentrated in the right side of the lungs.

The GradCAM visualizations elucidated on the transformative impact of feature fusion operations within our model. As observed in Figure 5, prior to the application of the global average pooling (GAP) and dense layers, the convolutional and multiplication layers within the feature fusion phase demonstrated a focused attention on regions that are clinically significant for pneumonia detection. These visualizations display well-defined areas within the chest X-rays, and they corresponded to the anatomical landmarks that are typically associated with pneumonia manifestations.

**Figure 6:**
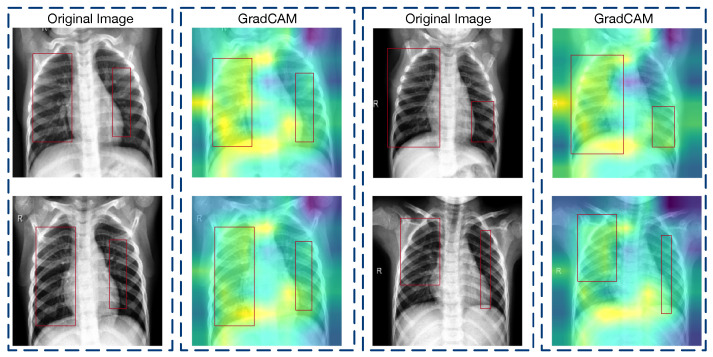
GradCAM heatmaps after feature fusion, where the changes in the attention phase’s post-reshape operations are visualized. Activation areas are more diffuse, but cover more important lung areas.

In stark contrast, Figure 6 presents the GradCAM output post-reshape operations, specifically those that highlighted the changes post-feature fusion and within the attention augmentation phase. The heatmaps exhibit a marked dispersion of attention, with the model’s focus appearing as abstract, pointillistic regions that do not conform to the structured patterns seen in the previous phase. This change indicated a potential loss of spatial fidelity, where the precise location of the relevant features became less distinct.

These visualizations suggest that, while the feature fusion strategy is successful in condensing and combining information from multiple channels, it may also abstract away the spatial specificity of pathologically relevant features. The consequent representations, despite being rich in aggregated information, may not retain the full context required for accurate pneumonia localization.

However, we also found that although the fused GradCAM heat map was less concentrated in activation regions than the pre-fusion one, its distribution covered more of the pathological regions that are useful for determining pneumonia—a testament to the benefits derived from integrating two distinct feature sets. This preservation and enrichment of relevant details form the cornerstone of our GradCAM analytical approach.

Consequently, we pinpointed the first convolutional layer following feature reshaping as the focus for our GradCAM analysis, whereby we aimed to underscore the distinctions in GradCAM visualizations across varying conditions.

**Figure 7:**
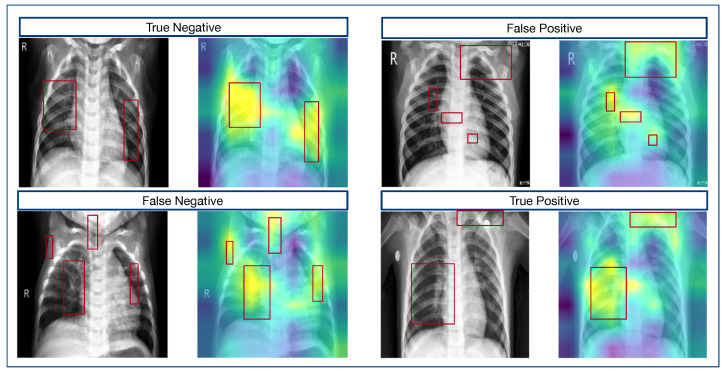
GradCAM visualization of the first convolutional layer following feature reshaping across varying conditions.

As is shown in Figure 7, in instances of accurate predictions (i.e., true negative and true positive cases), the activation regions highlighted in yellow were predominantly concentrated on the pathological areas that are indicative of pneumonia. This targeted activation lays the groundwork for the model’s accurate diagnostic decisions. Notably, while the true positive example exhibited some activation in non-relevant regions, the primary focus remained on the lung areas, which is crucial for pneumonia identification.

Conversely, when examining the instances of misclassification (i.e., the false negative and false positive cases), the GradCAM heatmaps, despite highlighting the areas associated with pneumonia, also extended their attention to peripheral, non-relevant regions, thus resulting in a more scattered pattern of activation. This diffusion of focus suggests that while the model generally identifies areas of interest, it may also erroneously prioritize regions unrelated to pneumonia pathology, thus leading to diagnostic inaccuracies.

Overall, the GradCAM analysis presented herein revealed that while the feature reshaping process inherent in our feature fusion component does lead to some spatial information loss, its judiciously chosen design parameters can mitigate this effect. Furthermore, the fusion of bidirectional features serves to partially offset the spatial detail loss incurred by reshaping. Despite the inherent limitations of our method—namely its inability to fully circumvent the loss of spatial information—it nonetheless exhibits significant interpretability.

#### 5.2.2. Potential Overuse of Attention Mechanisms

The second limitation concerns the model’s extensive application of attention mechanisms at various stages, i.e., from feature extraction to classification. While attention mechanisms such as multi-head and cross-attention are designed to enhance the model’s focus on relevant features, their overuse might result in a form of ’attention redundancy’. This redundancy could lead to the model overly specializing on certain features at the cost of a broader contextual understanding, which is critical for identifying all of the instances of the condition.

A closer look at the recall performance within our ablation study revealed a complex relationship between the attention mechanisms and the model’s sensitivity. While the recall improved when certain attention components were removed, suggesting that a more straightforward model without these mechanisms might capture positive cases more effectively, the removal of all attention components resulted in a significant drop in recall. This paradoxical outcome suggests that while attention mechanisms contribute to the model’s overall performance, an optimal balance is necessary to avoid diminishing the recall.

Comparative studies further illustrate this point. Models that do not employ attention mechanisms, such as those proposed by Bhatt [35] and traditional architectures like VGG, exhibit higher recall rates. The methods used by Wang [28] utilized a relatively simple attention mechanism. This observation implies that while attention mechanisms can enhance overall model performance, they may also, if not finely tuned, lead to a reduction in recall.

To quantify this aspect, let us consider Table 7, which highlights recall performances across different models and examines where the proposed model stands in relation to models that employ fewer or no attention mechanisms.

The data presented in the table suggests that models with a reduced number of attention mechanisms or those with none at all, such as VGG and the model proposed by Bhatt and Wang, have higher recall rates than our proposed model with multiple attention mechanisms. When attention mechanisms were reduced in our model, there was an apparent increase in recall, thus indicating that the attention mechanisms—while beneficial for certain aspects of performance—might indeed be limiting the model’s ability to capture all positive cases. However, the significant drop in recall observed when all of the components were removed confirms that some level of attention is beneficial and necessary for optimal model performance.

In summary, while attention mechanisms are powerful tools for feature enhancement, their overuse may detract from the recall performance, thus suggesting a need for the careful calibration of attention within the model to achieve a balance that maximizes sensitivity without sacrificing the gains in specificity and precision.

### 5.3. Future Work

One dimension of our future work will address the scalability of our model. Large datasets often come with increased intra-class variation and inter-class similarity, which poses a challenge to model performance. We intend to methodically enrich our training data with a multitude of imaging conditions and annotations. By embracing a wider spectrum of data, including rare and common variants of pathological findings, we aim to push the boundaries of the model’s robustness and sensitivity. This comprehensive scalability analysis will involve not just quantitative expansions, but also qualitative enrichment to create a model that is well versed in the nuances of complex clinical presentations.

Distinguishing between various pneumonia etiologies is a nuanced task requiring high-resolution insights. In our subsequent efforts, we will advance the model’s classification finesse to discern between bacterial, viral, and fungal pneumonia—each with distinct radiographic features and clinical implications. This approach will necessitate a layered strategy, where the model is trained with a more detailed categorization of pneumonia subtypes and may possibly integrate radiomics and other omics data. Furthermore, we envision a multimodal model that synergizes radiographic imaging with patient clinical metadata, thereby encapsulating a holistic view of the disease. Such a model would not only serve as a diagnostic aid, but also as a decision support system, thereby offering valuable predictive analytics for personalized treatment plans.

In addition, the versatility of our proposed model’s architecture, which is characterized by its advanced feature extraction and attention mechanisms, holds promise for a wide range of image-based classification tasks beyond pneumonia detection in chest X-ray images. Its capability in discerning intricate patterns makes it suitable for diverse applications, from satellite imagery analysis to wildlife monitoring, where fine-grained detail recognition is crucial.

The model’s modular design facilitates adaptability as it allows components like the EfficientNet and DenseNet backbones to be fine tuned or replaced to align with the specific demands of different domains. Moreover, the customizable attention mechanisms can be calibrated to focus on domain-specific features, thus enhancing the model’s precision and applicability.

Moving forward, we plan to broaden the scope of our model’s deployment, which will be achieved by rigorously testing and refining it on various datasets and exploring domain-specific architectural enhancements. This endeavor will ensure the model’s robust performance across a spectrum of image classification tasks, thereby marking a significant stride in the application of deep learning in diverse fields.

## 6. Conclusions

In this study, we introduced a sophisticated deep convolutional neural network that integrates the robust capabilities of EfficientNetB0 and DenseNet121 to accurately classify pneumonia from chest X-ray images. Our innovative approach, which is characterized by a cross-channel attention-based feature fusion and a series of attention mechanisms (including multi-head self-attention, attention augmentation, and dynamic pooling), has substantially enhanced the model’s diagnostic precision. The performance metrics underscore the efficacy of our model by achieving an accuracy of 95.19%, a precision of 98.38%, a recall of 93.84%, an F1 score of 96.06%, a specificity of 97.43%, and an AUC of 0.9564. These results not only validate the model’s ability to discern critical medical conditions accurately, but they also highlight its potential as a reliable tool in enhancing pneumonia diagnosis and, ultimately, patient care.

## Figures and Tables

**Table 1:** Data augmentation techniques and parameters.

Method	Parameters
Rotation	±15
Shift	0.1
Shear	0.2
Zoom	0.2
Brightness	0.8–1.2
Fill Mode	‘nearest’

**Table 2:** Hyperparameter optimization results with search space and best value.

Hyperparameter	Search Space	Best Value
Batch size	8, 16, 32	8
Dropout	0.2~0.5	0.2377
l2_coff	$1\times 10^{-4}$ ~$1\times 10^{-2}$	$6.72\times 10^{-3}$
Learning rate	$1\times 10^{-5}$~$1\times 10^{-3}$	$7.47\times 10^{-5}$

**Table 3:** Test and cross-validation results of the model.

	Accuracy (%)	Precision (%)	Recall (%)	F1 (%)	Specificity (%)	AUC
Test result	95.19	98.38	93.84	96.06	97.43	0.9564
Cross validation	91.30±6.59	98.96±1.53	89.34±9.81	93.56±5.19	96.94±4.58	0.9314±0.0385

**Table 4:** Performance comparison of the proposed model with respect to baselines and ablated studies.

Model	Accuracy (%)	Precision (%)	Recall (%)	F1 (%)	Specificity (%)	AUC
**Proposed Model**	**95.19**	**98.38**	**93.84**	**96.06**	**97.43**	**0.9564**
Remove Feature Fusion	92.94	91.99	97.17	94.51	85.89	0.9153
Remove Attention Augment	93.42	91.64	98.46	94.93	85.04	0.9175
Remove Dynamic Pooling	83.81	79.79	99.23	88.45	58.11	0.7867
Remove Multi-head Attention	90.86	88.81	97.69	93.04	79.48	0.8858
Remove All Components	90.22	97.95	86.15	91.67	97.01	0.9158
EfficientNetB0	73.71	74.35	88.46	80.79	49.14	0.6881
DenseNet121	90.71	97.15	87.69	92.18	95.72	0.9171

**Table 5:** Comparative performance of the proposed model against established baseline models.

Model	Accuracy (%)	Precision (%)	Recall (%)	F1 (%)	Specificity (%)	AUC
**Proposed Model**	**95.19**	**98.38**	**93.84**	**96.06**	**97.43**	**0.9564**
EfficientNetB0	73.71	74.35	88.46	80.79	49.14	0.6881
DenseNet121	90.71	97.15	87.69	92.18	95.72	0.9171
VGG16	88.62	84.74	99.74	91.63	70.08	0.8491
ResNet50	91.18	94.66	91.02	92.81	91.45	0.9123
InceptionV3	89.74	97.1	86.15	91.3	95.72	0.9094
MobileNetV3	60.09	61.92	93.84	74.61	3.84	0.4884

**Table 6:** Comparative results for the recent methods on the test set of the chest X-ray dataset.

Model	Accuracy (%)	Precision (%)	Recall (%)	F1 (%)
**Proposed Model**	**95.19**	**98.38**	**93.84**	**96.06**
Sharma, 2023 [34]	92.15	94.28	93.08	93.70
Bhatt, 2023 [35]	84.12	80.04	99.23	88.56
Goyal, 2023 [25]	94.31	88.89	95.41	92.03
Mabrouk, 2022 [36]	93.91	93.96	92.99	93.43
Wang, 2022 [28]	92.80	92.60	96.20	94.30

**Table 7:** Recall performance comparison and attention mechanism analysis.

Model	Recall (%)	Attention Mechanisms
Proposed Model	93.84	Multiple
Proposed Model without Feature Fusion	97.17	Reduced
Proposed Model without Attention Augment	98.46	Reduced
Proposed Model without Multi-head Attention	97.69	Reduced
Proposed Model without All Components	86.15	None
VGG (Baseline)	99.74	None
Bhatt, 2023 [35]	99.23	None
Wang, 2022 [28]	96.20	SE

## Data Availability

The data for this study come from publicly available datasets (https://www.kaggle.com/datasets/paultimothymooney/chest-xray-pneumonia/) from [32] (accessed on 6 December 2023).
